# Which chart and which cut-point: deciding on the INTERGROWTH, World Health Organization, or Hadlock fetal growth chart

**DOI:** 10.1186/s12884-021-04324-0

**Published:** 2022-01-10

**Authors:** Jessica Liauw, Chantal Mayer, Arianne Albert, Ariadna Fernandez, Jennifer A. Hutcheon

**Affiliations:** 1 Department of Obstetrics and Gynecology, University of British Columbia, C420-4500 Oak Street, BC Women’s Hospital, Vancouver, BC V6H 3N1 Canada; 2 Women’s Health Research Institute, BC Women’s Hospital and Health Centre, Room H214-F - 4500 Oak Street (Box 42), Vancouver, BC V6H 3N1 Canada

**Keywords:** Fetal growth, Growth charts, Fetal weight

## Abstract

**Abstract:**

**Objective:**

To determine how various centile cut points on the INTERGROWTH-21st (INTERGROWTH), World Health Organization (WHO), and Hadlock fetal growth charts predict perinatal morbidity/mortality, and how this relates to choosing a fetal growth chart for clinical use.

**Methods:**

We linked antenatal ultrasound measurements for fetuses > 28 weeks’ gestation from the British Columbia Women’s hospital ultrasound unit with the provincial perinatal database. We estimated the risk of perinatal morbidity/mortality (decreased cord pH, neonatal seizures, hypoglycemia, and perinatal death) associated with select centiles on each fetal growth chart (the 3rd, 10th, the centile identifying 10% of the population, and the optimal cut-point by Youden’s Index), and determined how well each centile predicted perinatal morbidity/mortality.

**Results:**

Among 10,366 pregnancies, the 10th centile cut-point had a sensitivity of 11% (95% CI 8, 14), 13% (95% CI 10, 16), and 12% (95% CI 10, 16), to detect fetuses with perinatal morbidity/mortality on the INTERGROWTH, WHO, and Hadlock charts, respectively. All charts performed similarly in predicting perinatal morbidity/mortality (area under the curve [AUC] =0.54 for all three charts). The statistically optimal cut-points were the 39th, 31st, and 32nd centiles on the INTERGROWTH, WHO, and Hadlock charts respectively.

**Conclusion:**

The INTERGROWTH, WHO, and Hadlock fetal growth charts performed similarly in predicting perinatal morbidity/mortality, even when evaluating multiple cut points. Deciding which cut-point and chart to use may be guided by other considerations such as impact on workflow and how the chart was derived.

**Supplementary Information:**

The online version contains supplementary material available at 10.1186/s12884-021-04324-0.

## Background

Despite the limitations of using size to identify fetal growth restriction [1], plotting estimated fetal size on a growth chart is one of the first steps in identifying which fetuses may benefit from closer monitoring in most jurisdictions. Recently, new fetal growth charts [2–4] have been derived from large prospective studies that performed serial ultrasound measurements of fetal growth in healthy pregnant women who were free of risk factors for fetal growth restriction. While some international guidelines recommend these new charts [1], others recommend using conventional population-based reference charts (e.g. Hadlock) [5]. In a recent commentary, leaders in the field emphasized the need for more evidence to guide recommendations on which growth charts to use, concluding that “ideally a comparison of diagnostic accuracy, or misclassification rates, of small-for-gestational-age and large-for-gestational-age fetuses in relation to morbidity and mortality using different criteria is necessary to make recommendations and remains an important data gap” (page S64) [6].

Previous studies linking estimated fetal weight centiles on these charts with adverse outcomes have only evaluated the charts’ predictive ability at the 10th or 5th centiles [7–11]. This is an important limitation because more extreme cut-points may be needed on the new charts because they reflect patterns of growth under optimal conditions [6].

We aimed to estimate the risks of adverse perinatal outcomes across the continuum of estimated fetal weight centiles on the INTERGROWTH-21st (INTERGROWTH), World Health Organization (WHO), and Hadlock fetal growth charts in a large ultrasound cohort linked with population-based perinatal outcome records, and to determine the abilities of various cut-points on each chart to distinguish fetuses that ultimately have perinatal morbidity/mortality. We also aimed to demonstrate a practical, evidence-based approach to selecting a fetal growth chart and centile cut-point for clinical use.

## Methods

### Study population

We included singleton fetuses who received an outpatient obstetrical ultrasound at > 28 weeks’ gestation at the British Columbia (BC) Women’s Hospital, in Vancouver, Canada, from April 1, 2000 to March 31, 2011. BC Women’s Hospital is a tertiary care teaching hospital with an annual delivery volume of approximately 7000 births. Obstetrical ultrasound records were linked with perinatal outcome data contained in the BC Perinatal Data Registry [12], a quality-controlled, previously validated [13] population database containing abstracted medical records for > 99% of births in BC [13]. Linkage with this provincial database enabled us to obtain birth outcome information on fetuses that had an ultrasound at BC Women’s Hospital, but delivered elsewhere in the province. We excluded multiple births, major congenital anomalies [14], and pregnancy terminations. Our analysis was restricted to fetuses with a last estimated fetal weight at or beyond 28 weeks, because fetal size differences at earlier gestations may have more heterogenous underlying etiologies [15]. We excluded fetuses with an implausibly small measurement for any biometric value, defined as <− 5 standard deviations (SD), based on commonly-used thresholds for flagging implausible pediatric growth values [16]. We assessed baseline characteristics of both our population and those of all non-anomalous, singleton births in BC > 28 weeks’ gestation to provide context for our findings.

### Classification of fetal growth

Ultrasound assessment of fetal size at the BC Women’s Hospital was performed by sonographers certified in obstetrical ultrasound. All ultrasound images were stored and verified by a Maternal Fetal Medicine or Radiology physician. Measurements were taken twice and averaged. Estimated fetal weight was calculated using a Hadlock formula that combines head circumference, abdominal circumference, and femur length [17]. The most recent measurement prior to delivery was used. We evaluated the accuracy of our estimated fetal weight measurements against neonatal birth weight among those who had their last ultrasound within 3 days of birth by calculating the mean percent difference ([estimated fetal weight- birthweight]/birthweight × 100) and the proportion of fetuses with an percent difference < 10%.

The ultrasound estimates of fetal weight for each fetus in our cohort were converted into gestational age-specific centiles using the previously published INTERGROWTH [2, 18], WHO [3], and Hadlock [19] fetal growth charts. We used exact gestational weeks in accordance with the charts’ intended use. We did not assess centiles on the recent Eunice Kennedy Shriver National Institute of Child Health and Human Development (NICHD) chart [4] or other customized charts [20] since we had no information on race/ethnicity and our population has a high proportion of mixed-ethnicity unions [21], which creates pragmatic challenges for using race/ethnicity-specific charts in our population. As the WHO and Hadlock growth charts did not allow for the calculation of exact percentiles, we estimated these by linear interpolation between published percentile values using previously recommended methods [22]. Estimated fetal weights more extreme than the upper and lower limits published on the chart (97.5 and 2.5 percentiles, respectively) were assigned the value of 99th percentile or 0.1st centile. These extreme assigned centiles would not have led to misclassification because the most extreme threshold examined in our study was the 3rd centile. Since the oldest gestational age on the WHO and Hadlock charts was 40 weeks, pregnancies in which the last estimated fetal weight measurement was taken after 40 weeks and 6 days’ (40 + 6) gestation were excluded (*n* = 43). Gestational age was determined by early ultrasound (< 20 weeks’ gestation), in accordance with current national guidelines [23].

### Outcomes

Our primary outcome was a composite of adverse perinatal outcomes commonly associated with fetal growth restriction (i.e., perinatal morbidity/mortality), defined as the occurrence of any of: arterial cord pH < 7.1, neonatal seizures (International Classification of Diseases, Tenth Revision, Clinical Modification [ICD-10-CM] codes 10: P90, 9: 779.0), hypoglycemia (ICD-10-CM codes 10: P703 and P704:, 9: 775.6), stillbirth (fetal death > 20 weeks’ gestation), or neonatal death (within 28 days after birth). Our secondary outcome was caesarean delivery for abnormal fetal heart rate tracing.

### Statistical analyses

We used descriptive statistics (means and standard deviations, frequencies with percentages) to summarize the maternal-fetal characteristics of our study population, and that of all non-anomalous singleton births in British Columbia delivered at or beyond 28 weeks’ gestation.

We first assessed how well each growth chart fit our population by determining the proportion of fetuses in low-risk pregnancies that were classified as less than the 3rd, 10th, and greater than the 90th and 97th centiles. Low-risk was defined as fetuses who ultimately delivered between 37 + 0 and 41 + 6 weeks’ gestation, were liveborn, had a maternal body mass index (BMI) between 18.5 and 25 kg/m^2^, had no maternal smoking during pregnancy, and had no maternal diabetes or hypertensive disorders of pregnancy. We only used low-risk pregnancies within our cohort for this portion of the analyses since the fetal growth charts we examined are intended to reflect a low risk population. For the remainder of the analyses below, we used the entire cohort of pregnancies presenting for ultrasound at our unit.

We estimated the association between fetal weight centiles and adverse perinatal outcomes using generalized additive modelling. Fetal weight centiles were modelled using a restricted cubic spline to allow smooth, non-linear patterns in risk as we hypothesized that risks may be increased at both low and high centiles [24]. Using these smoothed equations, we estimated the absolute risks of our outcomes at select centiles, and calculated the increases in risk compared with those at the 50th centile on each chart. We used bootstrapping (*n* = 10,000 replicates) to calculate the 95% confidence intervals for our estimates of absolute risk and absolute risk differences at each cut-point.

We assessed the ability of the INTERGROWTH, WHO, and Hadlock fetal growth charts to distinguish fetuses with perinatal morbidity/mortality by calculating the area under the receiver operating characteristic curve, and the sensitivity, specificity, positive predictive value, and negative predictive value, with 95% confidence intervals at select centile cut-points on each chart. We used Youden’s Index to define the statistically optimal centile cut-point to distinguish fetuses with adverse outcomes [25].

### Sensitivity analyses

Using each of the INTERGROWTH, WHO, and Hadlock fetal growth charts, we assessed the association between estimated fetal weight and *severe* perinatal morbidity/mortality, defined as the occurrence of any of: stillbirth, neonatal death, or neonatal seizures. For the WHO and INTERGROWTH charts, we repeated analyses for our primary outcome using centiles derived from fetal abdominal circumference (AC) measurements alone, rather than estimated fetal weight. We did not perform this sensitivity analysis with the Hadlock chart as AC reference curves were not available in the same publication as the estimated fetal weight curves.

This study was approved by the University of British Columbia/BC Children’s & Women’s Hospital Research Ethics Board, Certificate # H17–00798.

## Results

There were 10,366 pregnancies with feasible biometric measurements at > 28 weeks’ gestation, and which met our inclusion criteria (flow of participants shown in Fig. S1). Characteristics of the study cohort are described in Table 1. The median gestational age at the latest ultrasound > 28 weeks which assessed fetal growth was 34.3 weeks (Interquartile range [IQR] 31.9 to 36.6), and the mean gestational age at delivery was 38.1 weeks’ gestation (SD 2.2). As expected, our tertiary site cohort had a moderately higher risk profile than the general BC population (Table S1 and S2).

**Table 1:** Description of the cohort: singleton births > 28 weeks’ gestation, without major anomalies, with ultrasound at British Columbia Women’s Hospital, April 1, 2000 to March 31, 2011

Maternal-fetal characteristic	Births with ultrasound measurements at BC Women’s Hospital Mean ± SD or n(%) ***N*** = 10,366
Maternal age, years	33.0 ± 5.3
Nulliparous	5156 (49.7)
Diabetes	
Gestational diabetes	1373 (13.2)
Pre-existing diabetes	147 (1.4)
Hypertension	
Gestational hypertension	472 (4.6)
Pre-existing hypertension	221 (2.1)
Superimposed preeclampsia	43 (0.4)
Pre-eclampsia/ HELLP syndrome/ Eclampsia (de novo or superimposed on pre-existing hypertension)	253 (2.4)
Caesarean delivery	3736 (36.0)
Gestational age at latest scan, completed weeks median [IQR]	34.3 [31.9, 36.6]
Gestational age at delivery, completed weeks^a^	38.1(±2.2)
> 41 weeks’ gestation	93 (0.9)
Female sex	5138 (49.6)
Birthweight (grams)	3246.3 (±622.0)
Birthweight <10th%ile^b^	1164 (11.0)
APGAR score at 5 min < 7	143 (1.4)

^a^Missing data for *n* = 5 among pregnancies with ultrasound measurements at BC Women’s Hospital

^b^Based on a British Columbia population reference [29]

Among 840 births with an ultrasound < 3 days prior to delivery, the average percent difference between the ultrasound estimated fetal weight and birth weight was + 3.3% (SD 12.1), and 67% had an absolute difference that was within ±10% of their actual birthweight.

### Comparison of the estimated fetal weight distribution of our cohort to the charts’ distributions

**Fig. 1:**
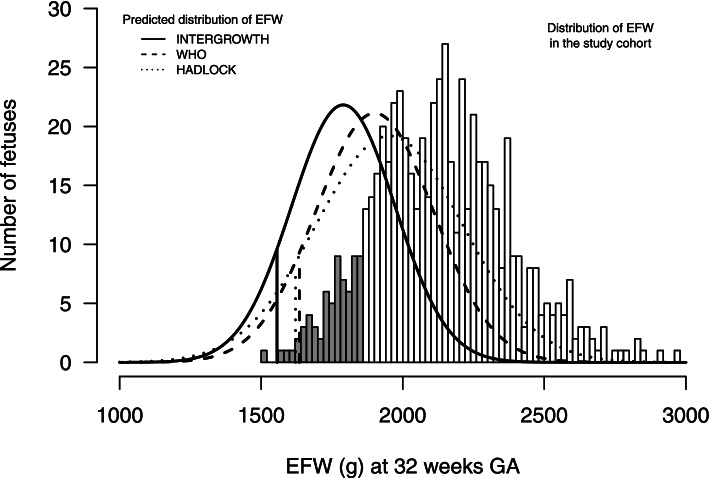
Comparison of observed estimated fetal weight (EFW) at 32 weeks’ gestation in the study cohort (bars), with reference values from the INTERGROWTH-21st chart (solid line), the World Health Organization (WHO) chart (dashed line), and the Hadlock chart (dotted line) . The grey bars indicate observations below the 10th centile in the study cohort. Vertical solid, dashed, and dotted lines indicate the 10th centile of the INTERGROWTH-21st, WHO, and Hadlock distributions

The estimated fetal weight distribution of the INTERGROWTH chart was shifted to systematically lower values than the distribution of low-risk pregnancies in our cohort (Fig. 1). For example, the INTERGROWTH chart identified 0.5% of our low-risk pregnancies as <3rd percentile and 2.0% as being <10th percentile, while 36.0 and 18.0% were > 90th and 97th percentiles, respectively. The WHO and Hadlock charts were also shifted to systematically lower values, albeit slightly less pronounced. On the WHO chart, 0.5 and 3.2% of low-risk pregnancies were < 3rd and 10th percentiles, and 33.5 and 15.2% were > 90th and 97th percentiles, respectively. On the Hadlock chart 1.8 and 5.8% of our low-risk pregnancies were < 3rd and 10th percentiles, and 16.8 and 10.4% as >90th and 97th percentiles, respectively.

### Association between estimated fetal weight centiles and adverse perinatal outcomes

**Fig. 2:**
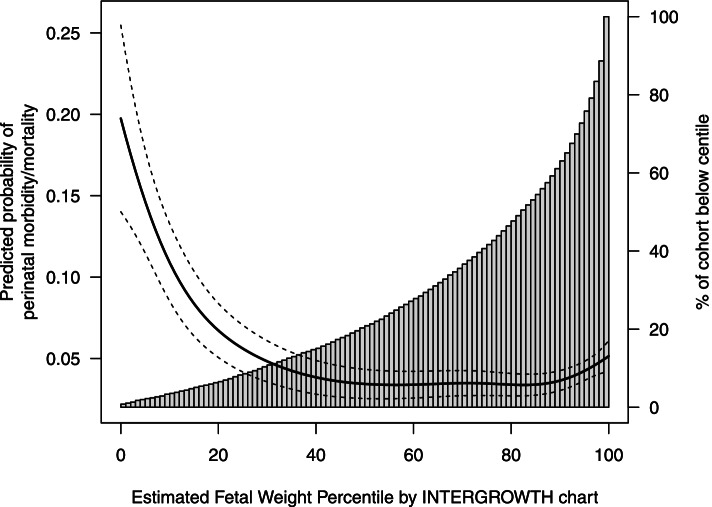
Predicted absolute risks of perinatal morbidity/mortality by estimated fetal weight centile (left side y-axis) and percent of the study cohort below each estimated fetal weight centile (right side y-axis) using the INTERGROWTH-21st chart

**Fig. 3:**
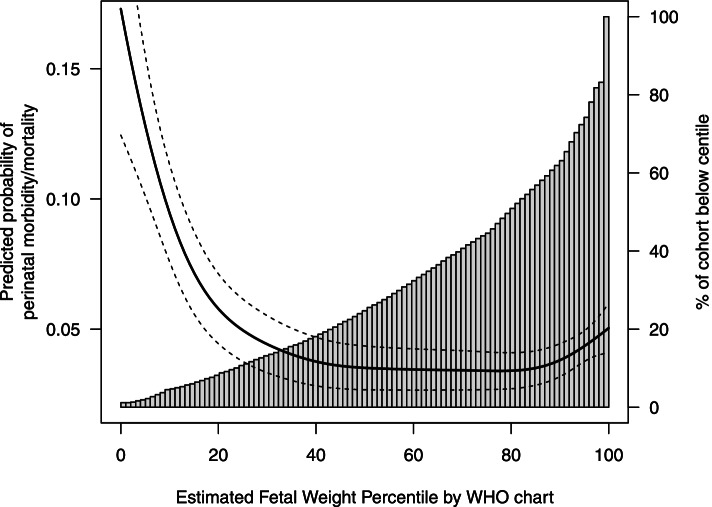
Predicted absolute risks of perinatal morbidity/mortality by estimated fetal weight centile (left side y-axis) and percent of the study cohort below each estimated fetal weight centile (right side y-axis) using the World Health Organization (WHO) chart

**Fig. 4:**
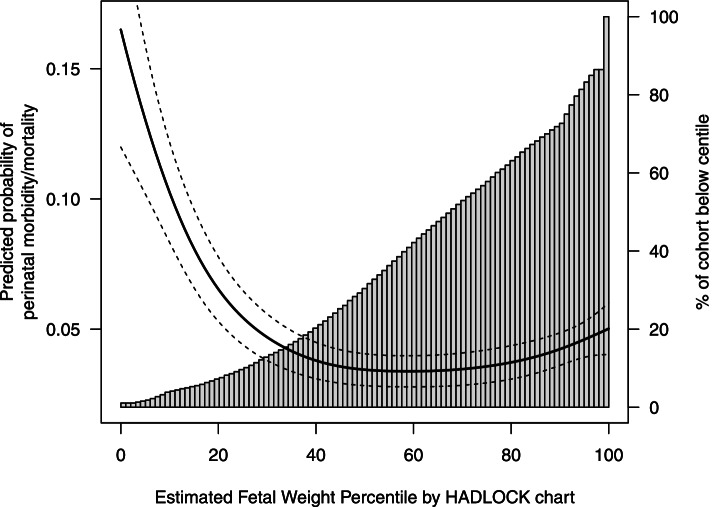
Predicted absolute risks of perinatal morbidity/mortality by estimated fetal weight centile (left side y-axis) and percent of the study cohort below each estimated fetal weight centile (right side y-axis) using the Hadlock chart

As shown in Table 2, 4.6 per 100 pregnancies in our cohort had our perinatal morbidity/mortality composite outcome. As expected, there were higher risks of perinatal morbidity/mortality at low and high estimated fetal weight percentiles (Figs. 2, 3 and 4). Because the INTERGROWTH distribution was further left-shifted than the WHO and Hadlock distributions, the 10th centile on the INTERGROWTH chart identified a smaller proportion of our cohort compared to the WHO and Hadlock charts (Tables 3, 4 and 5). Compared to the 50th centiles on each chart, those on the 10th percentile had 7.1 (95% CI 4.0 to 9.7), 5.5 (95% CI 2.8 to 7.7), and 6.6 (95% CI 4.4 to 8.9) excess cases of perinatal morbidity/mortality per 100 births, on the INTERGROWTH, WHO and Hadlock charts, respectively. Fetuses with estimated weights <10th percentile were 3.8 (95% CI: 2.8 to 4.9), 2.7 (95% CI: 2.1 to 3.4), and 2.9 (95% CI 2.3 to 3.7)-fold more likely to have perinatal morbidity/mortality compared with fetuses between the 10-90th percentiles, on the INTERGROWTH, WHO, and Hadlock charts, respectively.

**Table 2:** Incidence of perinatal morbidity/mortality in singleton births > 28 weeks’ gestation, without major anomalies, with ultrasound at British Columbia Women’s Hospital, April 1, 2000 to March 31, 2011

Perinatal health outcome	Births with ultrasound measurements at BC Women’s Hospital n(%) N = 10,366
Composite: one or more adverse neonatal morbidities	472 (4.6)
Stillbirth	26 (0.3)
Cord arterial pH < 7.1	206 (2.0)
Cord arterial pH missing or not obtained^a^	5049 (47.6)
Hypoglycemia	196 (1.8)
Neonatal seizures	24 (0.2)
Neonatal death	42 (0.4)
Any of: stillbirth, neonatal death, or neonatal seizures	91 (0.9)
Caesarean section for abnormal fetal heart rate	520 (5.0)

^a^ Umbilical cord blood gases were not routinely obtained in all deliveries during our study period

**Table 3:** INTERGROWTH-21st fetal growth chart centiles and perinatal morbidity/mortality: Proportion of the population below the cut-point, predicted absolute risks, and test performance characteristics

	Impact on workflow	Absolute risks		Test characteristics			
Cut-point centile	Proportion of population < centile cut-point, n(%)	Predicted absolute risk of morbidity/mortality, per 100 (95%CI)	Predicted absolute risk difference^a^, per 100 (95%CI)	Sensitivity % (95% CI)	Specificity % (95% CI)	Positive predictive value % (95% CI)	Negative predictive value % (95% CI)
3rd	139 (1.3)	16.6 (12.6, 21.0)	13.2 (9.1, 17.7)	6 (4, 9)	99 (99, 99)	22 (15, 29)	96 (95, 96)
10th	345 (3.3)	10.5 (7.6, 12.9)	7.1 (4.0, 9.7)	11 (8, 14)	97 (97, 97)	15 (11, 19)	96 (95, 96)
29th^b^	1022 (9.9)	5.1 (3.8, 6.6)	1.7 (0.1, 3.5)	21 (17, 25)	91 (90, 91)	10 (8, 12)	96 (96, 96)
39th^c^	1481 (14.3)	3.9 (2.8, 5.1)	0.5 (−0.7, 1.7)	26 (22, 30)	86 (86, 87)	8 (7, 10)	96 (96, 96)
50th	2107 (20.3)	3.4 (2.5, 4.4)	reference	31 (26, 35)	80 (79, 81)	7 (6, 8)	96 (96, 96)

^a^Compared with 50th centile. Calculated from 10,000 bootstrap replicates

^b^Centile that identifies 10% of the cohort as below that cut-point

^c^Statistically optimized cut-point by Youden’s Index

**Table 4:** World Health Organization fetal growth chart centiles and perinatal morbidity/mortality: Proportion of the population below the cut-point, predicted absolute risks, and test performance characteristics

	Impact on workflow	Absolute risks		Test characteristics			
Cut-point centile	Proportion of population < centile cut-point, n(%)	Predicted absolute risk of morbidity/mortality, per 100 (95%CI)	Predicted absolute risk difference^a^, per 100 (95%CI)	Sensitivity % (95% CI)	Specificity % (95% CI)	Positive predictive value % (95% CI)	Negative predictive value % (95% CI)
3rd	136 (1.3)	14.8 (11.2, 18.6)	11.3 (7.5, 15.3)	5 (3, 7)	99 (99, 99)	17 (11, 24)	96 (95, 96)
10th	466 (4.5)	9.0 (6.7, 11.0)	5.5 (2.8, 7.7)	13 (10, 16)	96 (95, 96)	13 (10, 16)	96 (95, 96)
24th^b^	1038 (10.0)	5.1 (3.8, 6.5)	1.6 (0.1, 3.2)	20 (17, 24)	90 (90, 91)	9 (8, 11)	96 (96, 96)
31st^c^	1401 (13.6)	4.5 (3.3, 5.9)	1.0 (−0.4, 2.8)	25 (21, 29)	87 (86, 88)	8 (7, 10)	96 (96, 96)
50th	2552 (24.7)	3.5 (2.6, 4.5)	reference	33 (29, 38)	76 (75, 77)	6 (5, 7)	96 (96, 96)

^a^Compared with 50th centile. Calculated from 10,000 bootstrap replicates

^a^Centile that identifies 10% of the cohort as below that cut-point

^b^Statistically optimized cut-point by Youden’s Index

**Table 5:** Hadlock fetal growth chart centiles and perinatal morbidity/mortality: Proportion of the population below the cut-point, predicted absolute risks, and test performance characteristics

	Impact on workflow	Absolute risks		Test characteristics			
Cut-point centile	Proportion of population < centile cut-point, n(%)	Predicted absolute risk of morbidity/mortality, per 100 (95%CI)	Predicted absolute risk difference^a^, per 100 (95%CI)	Sensitivity % (95% CI)	Specificity % (95% CI)	Positive predictive value % (95% CI)	Negative predictive value % (95% CI)
3rd	115 (1.1)	15.1 (11.1, 19.4)	11.6 (7.6, 16.1)	4 (3, 7)	99 (99, 99)	18 (12, 27)	96 (95, 96)
10th	398 (3.9)	10.1 (7.9, 12.2)	6.6 (4.4, 8.9)	12 (10, 16)	97 (96, 97)	15 (11, 18)	96 (95, 96)
26th^b^	1050 (10.2)	5.1 (3.9, 6.2)	1.7 (0.3, 2.9)	20 (17, 24)	90 (90, 91)	9 (7, 11)	96 (96, 96)
32st^c^	1450 (14.0)	4.4 (3.4, 5.5)	1.0 (−0.01, 2.5)	25 (21, 30)	86 (86, 87)	8 (7, 10)	96 (96, 96)
50th	3137 (30.4)	3.4 (2.7, 4.2)	reference	39 (34, 43)	70 (69, 71)	6 (5, 7)	96 (96, 96)

^a^Compared with 50th centile. Calculated from 10,000 bootstrap replicates

^b^Centile that identifies 10% of the cohort as below that cut-point

^c^Statistically optimized cut-point by Youden’s Index

The risk of Caesarean delivery for non-reassuring fetal heart rate tracing decreased exponentially as the estimated fetal weight reached approximately the 40th percentile, and did not change significantly thereafter (see Figs. S2, S3 and S4). Tables S3 to S5 show the absolute risks of caesarean delivery for non-reassuring fetal heart rate tracing associated with each centile cut-point on each chart. Fetuses measuring <10th percentile had 2.3 (95% CI: 1.7 to 3.1), 2.4 (95% CI: 1.9 to 3.0), and 2.4 (95% CI: 1.9 to 3.1)-fold increased risks of caesarean section for abnormal fetal heart tracing, compared to those at the 10-90th percentiles, on the INTERGROWTH, WHO, and Hadlock charts, respectively.

### Optimal cut-points

Test performance characteristics to detect perinatal morbidity/mortality at select centile cut-points for each chart are presented in Tables 3, 4 and 5. Overall, the ability of the charts to predict neonatal risk at the individual level was poor (AUC = 0.54, for each chart). At the traditional cut-point of the 10th centile, the sensitivity of the INTERGROWTH chart was 11% (95% CI: 8 to 14), and the positive predictive value was 15% (95% CI: 11 to 19). That is, use of this threshold on the INTERGROWTH chart would miss almost 90% of adverse perinatal outcomes, while approximately 85% of fetuses classified as ‘small for gestational age’ would have no adverse outcomes. Similarly, on the WHO chart, the 10th centile had a sensitivity of 13% (95% CI: 10 to 16), and on the Hadlock chart the 10th centile had a sensitivity of 12% (95%CI 10 to 16). Based on Youden’s Index, the optimal cut-points were the 39th, 31st, and 32nd centiles on the INTERGROWTH, WHO, and Hadlock charts, respectively. Even still, sensitivities and positive predictive values at these statistically optimized cut-points were poor.

Test performance characteristics to detect Caesarean delivery for abnormal fetal heart rate tracing at selected centile cut-points are presented in Tables S3 to S5. Again, the ability of each chart to predict individual-level risk of Caesarean delivery for non-reassuring fetal heart rate tracing was poor (AUC of 0.57 for INTERGROWTH and WHO, 0.56 for Hadlock). The optimal cut-point centiles were the 36th, 39th, and 29th centiles on the INTERGROWTH, WHO, and Hadlock charts, respectively.

### Sensitivity analyses

The association between estimated fetal weight centiles on the INTERGROWTH, WHO, and Hadlock fetal growth charts and the occurrence of severe perinatal morbidity/mortality (any of stillbirth, neonatal death, or neonatal seizures) (Figs. S5, S6 and S7), was not meaningfully different compared to our primary outcome. The optimal cut-points for identifying fetuses with severe perinatal morbidity/mortality, were the 21st, 31st, and 32nd centiles on the INTERGROWTH, WHO, and Hadlock charts, respectively. Likewise, conclusions were similar when using the abdominal circumference alone to determine the centiles, compared with using the estimated fetal weight (Figs. S8 and S9). Using abdominal circumference alone, the optimal cut-points for predicting our primary outcome were the 33rd and 31st centiles on the INTERGROWTH [2] and WHO [3] charts, respectively.

## Discussion

### Main findings

As expected, risks of adverse perinatal outcomes were higher at lower estimated fetal weight centiles on the INTERGROWTH, WHO, and Hadlock fetal growth charts. However, these increased population-level risks at lower centiles did not translate into accurate prediction of individual-level risk: sensitivity and positive predictive values across a range of centile cut-points on all three charts were similarly low. The optimal cut-point on each chart would have identified 13 to 14% of pregnancies in our cohort as ‘small for gestational age’, and even then, would have still missed over 70% of fetuses that ultimately had perinatal morbidity/mortality.

Our results are consistent with previous studies. Using estimated fetal weights of 9409 fetuses enrolled in the 1987–1991 RADIUS trial, the abilities of the INTERGROWTH, WHO, and the NICHD charts to predict adverse perinatal outcomes were similarly poor (AUCs between 0.50 and 0.59) [9]. A few smaller studies have evaluated the predictive abilities of these charts in more contemporary cohorts; primarily limited to analysis of the 10th centile. A study of 3437 fetuses of African-American women compared eight fetal growth charts, including INTERGROWTH, WHO and the NICHD charts. All charts poorly detected composite adverse perinatal outcomes at the 10th centile (e.g., AUC 0.55 for INTERGROWTH), but with a fixed false positive rate of < 10% the INTERGROWTH chart performed better than others for some perinatal outcomes, albeit with a sensitivity of only 22% [8]. Furthermore, in a cohort of 1054 women from St. Louis and Tampa, USA, the 10th centile on a customized standard and the INTERGROWTH standard performed poorly to identify adverse neonatal outcomes (AUC 0.52 for the customized chart, and AUC 0.51 for the INTERGROWTH chart) [7]. When this same cohort was used to compare the Hadlock and INTERGROWTH fetal growth charts, results were similar [11]. Our results extend previous work in this area by reporting chart performance across a wide range of centile cut-points other than just the 10th centile, using a cohort of fetuses that is, to our knowledge, larger than any other published cohort examining associations between estimated fetal weight and perinatal outcomes.

### Strengths and limitations

Strengths of our study include the large sample size and the use of antenatal ultrasound measurements. Furthermore, in addition to reporting test performance characteristics for multiple cut-points on each chart, we provide absolute risks associated with each centile examined, which can help clinicians and patients understand what it means to be on each centile. Finally, our access to outcome data for all fetuses scanned at our centre, not only those who delivered there, supports the generalizability of our findings. We used the most recent third trimester ultrasound performed at our centre for each participant in our analysis, which was, on average, 1 month before delivery. Fetuses may not have maintained the same centile between their last ultrasound and delivery, which may have contributed to the charts showing poorer performance. However, this limitation would have affected all charts assessed, and reflects an inherent limitation of using these charts in clinical practice. In addition, since our analysis included pregnancies that could have delivered at different centers across British Columbia, there may have been variations in management at each site in response to ultrasound findings, which could have impacted neonatal morbidity, e.g. earlier delivery leading to increased rates of hypoglycemia. However, none of the charts we assessed were in clinical use during our study period, so practice patterns based on ultrasound findings would have been unlikely to bias outcomes in favour of any of the charts we studied. Finally, there is no gold standard outcome which defines fetal growth restriction. Neonatal morbidities that may be the result of growth restriction, such as hypoglycemia, may also be the result of other complications such as prematurity. We attempted to select perinatal outcomes that are directly related to growth restriction, as opposed to prematurity, with the acknowledgement that these complications and their outcomes are often intertwined, and this could also make the charts’ performance seem poorer.

### Importance

From a practical standpoint, our demonstration of how the target proportion of cases to be detected and absolute risks of adverse perinatal outcomes change across centiles on multiple fetal growth charts can help clinicians and policy-makers choose a chart and cut-point for clinical practice while taking into account a unit’s workflow demands. Furthermore, knowing that the diagnostic accuracy of each of these charts is similarly poor may motivate clinicians and policy-makers to base selection of a chart and threshold centile on other factors, such as the methodology the chart was derived from (e.g., is it important that the WHO and INTERGROWTH charts were derived from a multi-ethnic populations, unlike Hadlock?), or consistency with other jurisdictions or research studies (e.g. is it important to adopt a chart that is used in clinical trials so research findings can be easily translated into clinical practice?).

Our findings suggest that fetal biometry alone is unlikely to be a sufficiently accurate predictor of perinatal outcome, and that the use of fetal doppler studies and other functional and/or serial assessments are needed to improve the detection of fetal growth restriction [26, 27]. In addition, a number of novel biomarkers for detection of fetal growth restriction have been identified, and future work to establish their predictive ability, alone and combination with fetal biometry and doppler assessments, is a high-priority for the field [28]. Since no one chart significantly outperformed the others in predicting adverse outcomes, we have decided to choose a fetal growth standard derived from a large, contemporary sample, which best fits the distribution of our population, acknowledging that choosing among even the best fitting charts means that we will identify less than 10% of our population as being <10th centile in estimated fetal weight. We also decided to choose a cut-point on this standard which will identify a proportion of our population for which we can feasibly increase fetal monitoring. This evidence-based approach to choosing a chart and cut-point will help to unify our definition of fetal size in our jurisdiction, which can optimize both clinical care and future research in fetal growth.

## Conclusion

The INTERGROWTH, WHO, and Hadlock fetal growth charts perform similarly to distinguish fetuses that will have adverse perinatal outcomes across multiple cut-point centiles. Decisions on which chart and cut-point to use can be made by considering the proportion of fetuses that will fall below a cut-point, the absolute risks associated with a cut-point, and the robustness of the methodology underlying various growth chart options.

## Supplementary Information

**Additional file 1.**

**Additional file 2.**

**Additional file 3.**

**Additional file 4.**

**Additional file 5.**

**Additional file 6.**

**Additional file 7.**

**Additional file 8.**

**Additional file 9.**

**Additional file 10.**

## Data Availability

The data that support the findings of this study are available from BC Perinatal Data Registry but restrictions apply to the availability of these data, which were used under license for the current study, and so are not publicly available. Data are however available from the authors upon reasonable request and with permission of BC Perinatal Data Registry.

## Abbreviations

AC: Abdominal Circumference
AUC: Area Under the Curve
BC: British Columbia
BMI: Body Mass Index
ICD-10-CM: International Classification of Diseases, Tenth Revision, Clinical Modification
INTERGROWTH: INTERGROWTH-21st
IQR: Interquartile Range
NICHD: Eunice Kennedy Shriver National Institute of Child Health and Human Development
SD: Standard Deviation
WHO: World Health Organization
