# Supplementary material for: Which chart and which cut-point: deciding on the INTERGROWTH, World Health Organization, or Hadlock fetal growth chart
Source: BMC Pregnancy Childbirth. 2022 Jan 10;22:25. doi: 10.1186/s12884-021-04324-0 (PMC8751336; doi:10.1186/s12884-021-04324-0)
Supplement: Supplementary file 1 — Additional file 1. [file 12884_2021_4324_MOESM1_ESM.docx]

**SUPPLEMENT**

**Table S1**. Description of the cohort, N=10,366, and all singleton births >28 weeks’ gestation, N=465,112, without major anomalies, in British Columbia, April 1, 2000 to March 31, 2011

| **Maternal-fetal characteristic** | **Pregnancies with ultrasound measurements at BC Women’s Hospital**  **mean± SD or n(%)** | **All British Columbia births**  **mean± SD or n(%)** |
| --- | --- | --- |
| N | 10,366 | 465,112 |
| Maternal age, years | 33.0 ±5.3 | 30.3 ±5.6 |
| Nulliparous* | 5287 (49.9) | 212,913 (45.8) |
| Diabetes |  |  |
| Gestational diabetes | 1396 (13.0) | 30,543 (6.6) |
| Pre-existing diabetes | 21 (0.9) | 4,045 (0.9) |
| Hypertension |  |  |
| Gestational hypertension | 486 (4.6) | 16,467 (3.5) |
| Pre-existing hypertension | 228 (2.1) | 2,518 (0.5) |
| Superimposed preeclampsia | 45 (0.4) | 372 (0.1) |
| Pre-eclampsia/ HELLP syndrome/ Eclampsia (de novo or superimposed on pre-existing hypertension) | 260 (2.5) | 5,450 (1.2) |
| Caesarean delivery | 3,819 (36.0) | 130,997 (28.1) |
| Gestational age at latest scan, completed weeks median [IQR] | 34.3 [31.9, 36.6] | Not applicable |
| Gestational age at delivery, completed weeks^ | 38.1(±2.2) | 38.8 (±1.7) |
| >41 weeks’ gestation | 97 (0.9) | 6,671 (1.4) |
| Female sex^#^ | 5,249 (49.5) | 226,483 (48.7) |
| Birthweight (grams) | 3244.6 (±623.3) | 3445.9 ±527.4 |
| Birthweight <10^th^%ile^†^ | 1,164 (11.0) | 31,615 (6.8) |
| APGAR score at 5 minutes <7 | 150 (1.4) | 6,847 (1.5) |

*Missing data for n=30 among all births in British Columbia.

^Missing data for n=5 among pregnancies with ultrasound measurements at BC Women’s Hospital.

^#^Missing data for n=7 among all births in British Columbia.

^†^Based on a British Columbia population reference.^29^

**Table S2.** Incidence of perinatal morbidity/mortality in births with ultrasound measurements at BC Women’s Hospital, N=10,366, and in all singleton births >28 weeks’ gestation (N=465,112) with no major congenital anomalies, in British Columbia, April 1, 2000 to March 31, 2011

| **Perinatal health outcome** | **Births with ultrasound measurements at BC Women’s Hospital**  **n(%)** | **Births in British Columbia**  **n(%)** |
| --- | --- | --- |
| N | 10,366 | 465,112 |
| Composite: one or more adverse neonatal morbidities | 484 (4.6) | 12,303 (2.6) |
| Stillbirth | 28 (0.3) | 267 (0.1) |
| Cord arterial pH <7.1 | 210 (2.0) | 6,696 (1.4) |
| Cord arterial pH missing or not obtained* | 5,049 (47.6) | 250,339 (53.8) |
| Hypoglycemia | 201 (1.9) | 4,316 (0.9) |
| Neonatal seizures | 24 (0.2) | 556 (0.1) |
| Neonatal death | 43 (0.4) | 943 (0.2) |
| Any of: stillbirth, neonatal death, or neonatal seizures | 94 (0.9) | 1,738 (0.4) |
| Caesarean section for abnormal fetal heart rate | 532 (5.0) | 18,424 (4.0) |

* Umbilical cord blood gases were not routinely obtained in all deliveries during our study period.

**Table S3.** INTERGROWTH-21^st^ fetal growth chart centiles and caesarean section for abnormal fetal heart rate tracing: Proportion of the population below the cut-point, predicted absolute risks, and test performance characteristics.

| **Impact on workflow** | | **Absolute risks** | | **Test characteristics** | | | |
| --- | --- | --- | --- | --- | --- | --- | --- |
| Cut-point centile | Proportion of population < centile cut-point, n(%) | Predicted absolute risk of caesarean for abnormal tracing per 100 (95%CI) | Predicted absolute risk difference^ per 100 (95%CI) | Sensitivity  %  (95% CI) | Specificity  %  (95% CI) | Positive predictive value  % (95% CI) | Negative predictive value  % (95% CI) |
| 3^rd^ | 139 (1.3) | 15.9  (11.9, 20.2) | 11.4  (7.1, 15.9) | 6  (4, 8) | 99  (99, 99) | 22  (15, 29) | 95  (95, 96) |
| 10^th^ | 345 (3.3) | 11.4  (8.8, 13.9) | 6.9  (4.2, 9.6) | 9  (7, 12) | 97  (97, 97) | 14  (10, 18) | 95  (95, 96) |
| 29th* | 1022 (9.9) | 6.0  (4.7, 7.5) | 1.5  (0.03, 3.28) | 21  (17, 24) | 91  (90, 91) | 10  (9, 13) | 96  (95, 96) |
| 36th** | 1345 (13.0) | 5.1  (3.8, 6.4) | 0.6  (-0.7, 1.8) | 24  (21, 28) | 88  (87, 88) | 9  (8, 11) | 96  (95, 96) |
| 50th | 2107 (20.3) | 4.5  (3.4, 5.5) | reference | 30  (26, 35) | 80  (79, 81) | 7  (6, 9) | 96  (95, 96) |

^Compared with 50^th^ centile. Calculated from 10,000 bootstrap replicates.

*Centile that identifies 10% of the cohort as below that cut-point.

**Statistically optimized cut-point by Youden’s Index.

**Table S4.** World Health Organization fetal growth chart centiles and caesarean section for abnormal fetal heart rate tracing: Proportion of the population below the cut-point, predicted absolute risks, and test performance characteristics.

|  | **Impact on workflow** | **Absolute risks** | | **Test characteristics** | | | |
| --- | --- | --- | --- | --- | --- | --- | --- |
| Cut-point centile | Proportion of population < centile cut-point, n(%) | Predicted absolute risk of caesarean for abnormal tracing, per 100 (95%CI) | Predicted absolute risk difference^,  per 100 (95%CI) | Sensitivity, %  (95% CI) | Specificity,  %  (95% CI) | Positive predictive value,  % (95% CI) | Negative predictive value,  % (95% CI) |
| 3^rd^ | 136 (1.3) | 15.9  (11.9, 19.9) | 11.6 (7.3, 15.8) | 6 (4, 8) | 99 (99, 99) | 22 (15, 30) | 95 (95, 96) |
| 10^th^ | 466 (4.5) | 9.6  (7.1, 11.9) | 5.4 (2.4, 7.8) | 13 (11, 17) | 96 (96, 96) | 15 (12, 18) | 95 (95, 96) |
| 24th* | 1038 (10.0) | 6.2  (4.8, 8.2) | 1.9 (0.2, 4.2) | 21 (17, 24) | 91 (90, 91) | 10 (9, 12) | 96 (95, 96) |
| 39^th**^ | 1838 (17.8) | 4.3  (3.0, 5.5) | 0.1 (-1.7, 1.2) | 29 (25, 33) | 83 (82, 84) | 8 (7, 10) | 96 (95, 96) |
| 50th^#^ | 2552 (24.7) | 4.3  (3.2, 5.5) | reference | 34 (30, 38) | 76 (75, 77) | 7 (6, 8) | 96 (95, 96) |

^Compared with 50^th^ centile. Calculated from 10,000 bootstrap replicates.

*Centile that identifies 10% of the cohort as below that cut-point.

**Statistically optimized cut-point by Youden’s Index.

**Table S5.** Hadlock fetal growth chart centiles and caesarean section for abnormal fetal heart rate tracing: Proportion of the population below the cut-point, predicted absolute risks, and test performance characteristics.

|  | **Impact on workflow** | **Absolute risks** | | **Test characteristics** | | | |
| --- | --- | --- | --- | --- | --- | --- | --- |
| Cut-point centile | Proportion of population < centile cut-point, n(%) | Predicted absolute risk of caesarean for abnormal tracing, per 100 (95%CI) | Predicted absolute risk difference^,  per 100 (95%CI) | Sensitivity, %  (95% CI) | Specificity,  %  (95% CI) | Positive predictive value,  % (95% CI) | Negative predictive value,  % (95% CI) |
| 3^rd^ | 115 (1.1) | 16.8 (12.6, 21.1) | 12.3 (8.1, 16.7) | 5 (3, 7) | 99 (99, 99) | 23 (15, 31) | 95 (95, 96) |
| 10^th^ | 398 (3.9) | 10.5 (7.9, 12.8) | 6.1 (3.0, 8.5) | 12 (9, 15) | 97 (96, 97) | 15 (12, 19) | 95 (95, 96) |
| 26th* | 1050 (10.2) | 6.0 (4.7, 8.1) | 1.6 (0.0, 3.7) | 21 (17, 24) | 90 (90, 91) | 10 (8, 12) | 96 (95, 96) |
| 29^th**^ | 1255 (12.1) | 5.7 (4.5, 7.5) | 1.2  (-0.2, 3.2) | 24 (20, 28) | 88 (88, 89) | 10 (8, 12) | 96 (95, 96) |
| 50th | 3137 (30.4) | 4.4 (3.6, 5.5) | reference | 40 (36, 44) | 70 (69, 71) | 7 (6, 8) | 96 (95, 96) |

^Compared with 50^th^ centile. Calculated from 10,000 bootstrap replicates.

*Centile that identifies 10% of the cohort as below that cut-point.

**Statistically optimized cut-point by Youden’s Index.

**Figure S1.** Participant inclusion flowchart

**Figure S2.** Predicted absolute risks of caesarean section for abnormal fetal heart rate tracing by estimated fetal weight centile (left side y-axis) and percent of the study cohort below each estimated fetal weight centile (right side y-axis) using the INTERGROWTH-21^st^ fetal growth chart

**Figure S3.** Predicted absolute risks of caesarean section for abnormal fetal heart rate tracing by estimated fetal weight centile (left side y-axis) and percent of the study cohort below each estimated fetal weight centile (right side y-axis) using the World Health Organization (WHO) fetal growth chart.

**Figure S4.** Predicted absolute risks of caesarean section for abnormal fetal heart rate tracing by estimated fetal weight centile (left side y-axis) and percent of the study cohort below each estimated fetal weight centile (right side y-axis) using the Hadlock fetal growth chart.

**Figure S5.** Predicted absolute risks of severe perinatal morbidity/mortality (stillbirth, neonatal seizures, or neonatal death) by estimated fetal weight percentile (left side y-axis) and percent of the study cohort below each estimated fetal weight percentile (right side y-axis) using the INTERGROWTH-21^st^ fetal growth chart.

**Figure S6** Predicted absolute risks of severe perinatal morbidity/mortality (stillbirth, neonatal seizures, or neonatal death) by estimated fetal weight centile (left side y-axis) and percent of the study cohort below each estimated fetal weight centile (right side y-axis), using the World Health Organization (WHO) fetal growth chart.

**Figure S7** Predicted absolute risks of severe perinatal morbidity/mortality (stillbirth, neonatal seizures, or neonatal death) by estimated fetal weight centile (left side y-axis) and percent of the study cohort below each estimated fetal weight centile (right side y-axis), using the Hadlock fetal growth chart.

**Figure S8.** Predicted absolute risks of perinatal morbidity/mortality by abdominal circumference centile (left side y-axis) and percent of the study cohort below each abdominal circumference centile (right side y-axis) using the INTERGROWTH-21^st^ fetal growth chart.

**Figure S9.** Predicted absolute risks of perinatal morbidity/mortality by abdominal circumference centile (left side y-axis) and percent of the study cohort below each abdominal circumference centile (right side y-axis) using the World Health Organization (WHO) chart.
