# Supplementary material for: Which chart and which cut-point: deciding on the INTERGROWTH, World Health Organization, or Hadlock fetal growth chart
Source: BMC Pregnancy Childbirth. 2022 Jan 10;22:25. doi: 10.1186/s12884-021-04324-0 (PMC8751336; doi:10.1186/s12884-021-04324-0)
Supplement: Supplementary file 2 — Additional file 2. [file 12884_2021_4324_MOESM2_ESM.docx]

**Figure S1.** Participant inclusion flowchart

Selected according to inclusion criteria

Analyzed

Identified

Singleton pregnancies with an ultrasound at BC Women’s Hospital from April 1, 2000 to March 31, 2011 and continuing beyond 20 weeks (n=39,422 with 65,528 ultrasounds)

Excluded pregnancies (n=28,817)

- Last available ultrasound <28 weeks (n=24,962)
- Last available ultrasound was >28 weeks but did not include valid biometry (n=2,825)
- Major congenital anomaly (n=1,269)

Eligible singleton pregnancies (n=10,366)

World Health Organization Fetal Growth Standard

(n=10,323)

INTERGROWTH-21^st^ fetal growth standard

(n=10,366)

Hadlock Fetal Growth Chart

(n=10,323)
