# Supplementary material for: Which chart and which cut-point: deciding on the INTERGROWTH, World Health Organization, or Hadlock fetal growth chart
Source: BMC Pregnancy Childbirth. 2022 Jan 10;22:25. doi: 10.1186/s12884-021-04324-0 (PMC8751336; doi:10.1186/s12884-021-04324-0)
Supplement: Supplementary file 5 — Additional file 5. [file 12884_2021_4324_MOESM5_ESM.pdf]

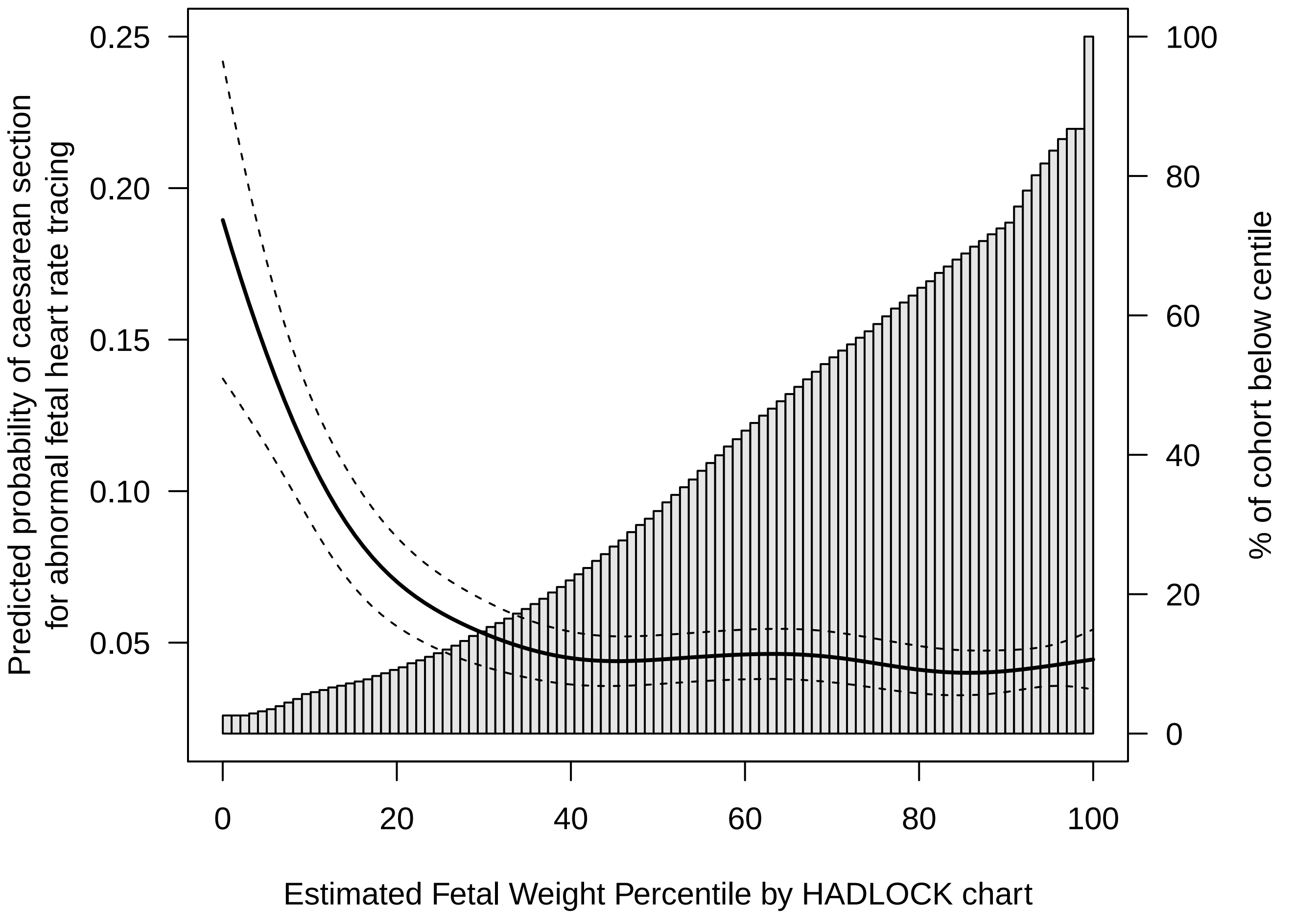
Predicted probability of caesarean section for abnormal fetal heart rate tracing
0.25
0.20
0.15
0.10
0.05
% of cohort below centile
100
80
60
40
20
0
0
20
40
60
80
100
Estimated Fetal Weight Percentile by HADLOCK chart
