# Supplementary material for: Which chart and which cut-point: deciding on the INTERGROWTH, World Health Organization, or Hadlock fetal growth chart
Source: BMC Pregnancy Childbirth. 2022 Jan 10;22:25. doi: 10.1186/s12884-021-04324-0 (PMC8751336; doi:10.1186/s12884-021-04324-0)
Supplement: Supplementary file 7 — Additional file 7. [file 12884_2021_4324_MOESM7_ESM.pdf]

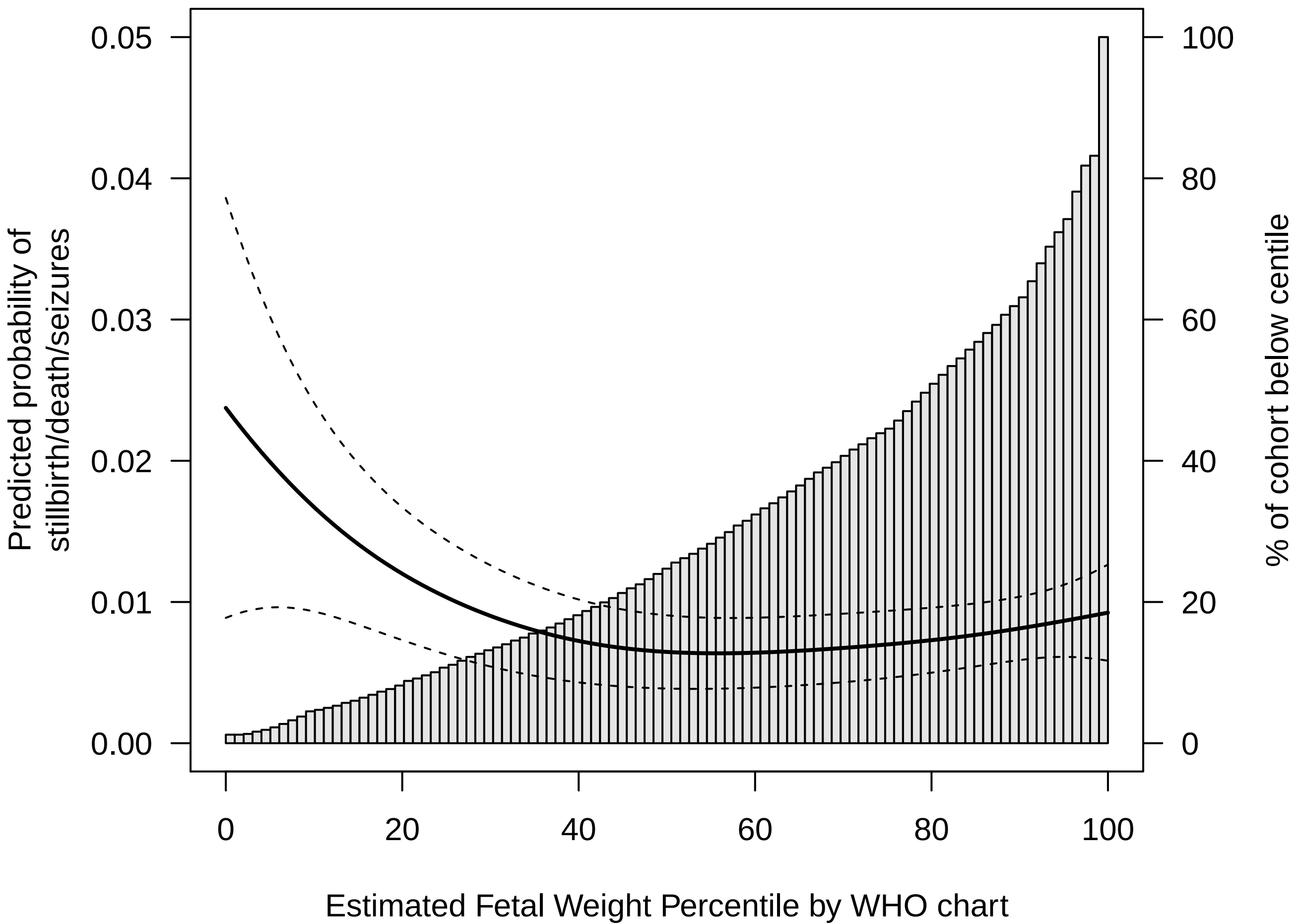

0.05
0.04
0.03
0.02
0.01
0.00
Predicted probability of stillbirth/death/seizures
100
80
60
40
20
0
% of cohort below centile
0
20
40
60
80
100
Estimated Fetal Weight Percentile by WHO chart
