# Supplementary material for: Which chart and which cut-point: deciding on the INTERGROWTH, World Health Organization, or Hadlock fetal growth chart
Source: BMC Pregnancy Childbirth. 2022 Jan 10;22:25. doi: 10.1186/s12884-021-04324-0 (PMC8751336; doi:10.1186/s12884-021-04324-0)
Supplement: Supplementary file 9 — Additional file 9. [file 12884_2021_4324_MOESM9_ESM.pdf]

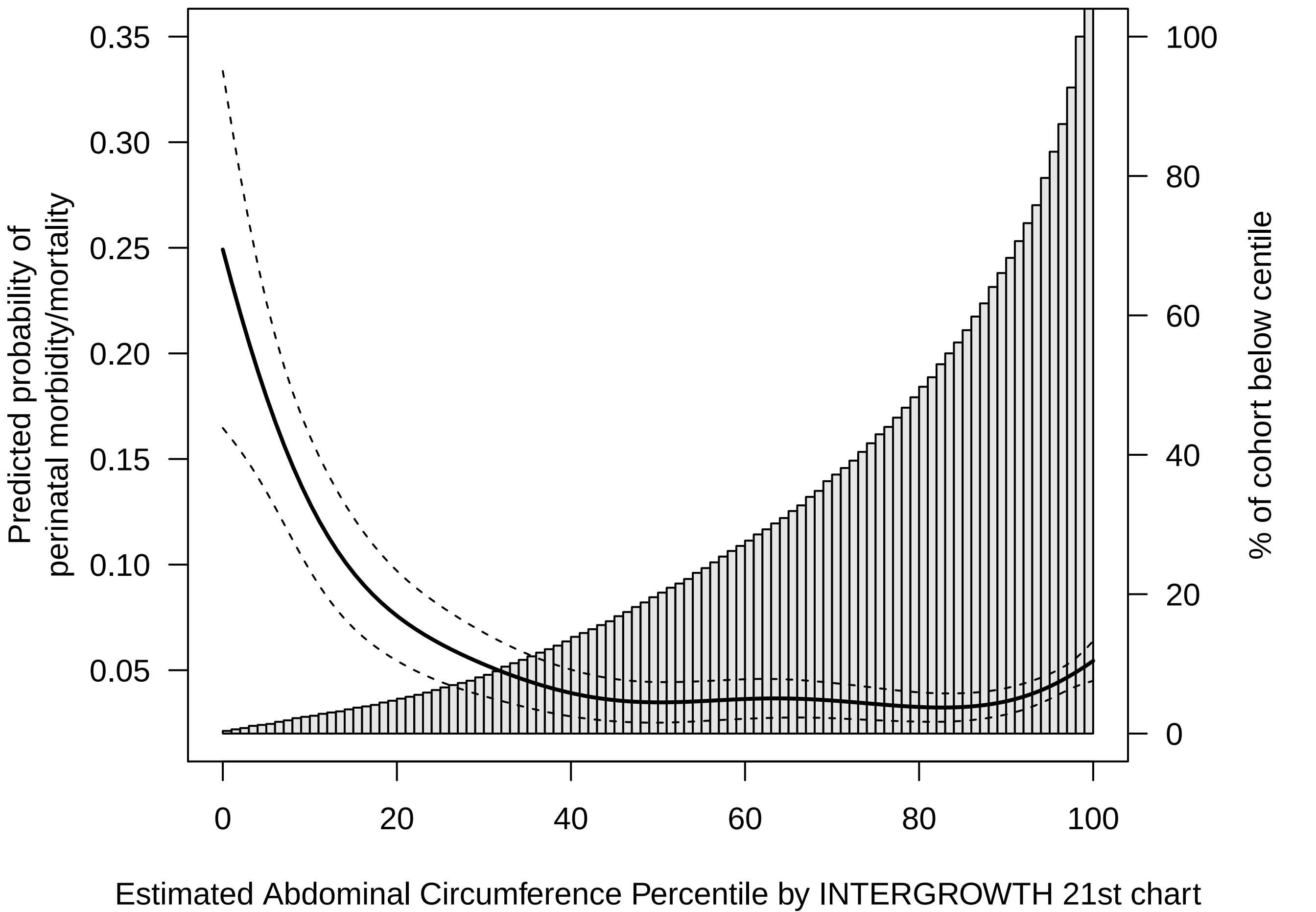
Predicted probability of
perinatal morbidity/mortality
0.35
0.30
0.25
0.20
0.15
0.10
0.05
0
20
40
60
80
100
% of cohort below centile
Estimated Abdominal Circumference Percentile by INTERGROWTH 21st chart
